# The Effect of Stress on Depression in Postgraduate Students: Mediating Role of Research Self-Efficacy and Moderating Role of Growth Mindset

**DOI:** 10.3390/bs15030266

**Published:** 2025-02-24

**Authors:** Tongji Li, Jun Guan, Yishu Huang, Xinglin Jin

**Affiliations:** 1 Institute of Vocational Education and Training, Tongji University, Shanghai 201804, China; litj@tongji.edu.cn (T.L.); 2231428@tongji.edu.cn (J.G.); 2430012@tongji.edu.cn (Y.H.); 2 School of Education Science, Jiangsu Normal University, Xuzhou 221116, China

**Keywords:** graduate students, stress, depression, growth mindset, research self-efficacy

## Abstract

This study investigates the relationship between stress and depression among postgraduate students, with a focus on the mediating role of research self-efficacy and the moderating role of growth mindset. A questionnaire survey was conducted among 2278 graduate students nationwide, utilizing the Mindset Scale, Depression–Anxiety–Stress Scale, and Research Self-Efficacy Scale. Data were analyzed using SPSS 27.0 and AMOS 24.0. The results indicate the following: (1) Stress has a significant positive relationship with depression, while stress is negatively related to research self-efficacy, and research self-efficacy is negatively related to depression. (2) Research self-efficacy partially mediates the relationship between stress and depression, accounting for 35.6% of the total effect. (3) Growth mindset moderates both the relationship between stress and research self-efficacy and the relationship between research self-efficacy and depression. These findings reveal the mechanisms through which stress affects depression among graduate students, underscore the importance of mental health education, and provide scientific evidence for universities and educational institutions to design targeted prevention and intervention measures.

## 1. Introduction

Postgraduate education plays a critical role in fostering advanced expertise, critical thinking, and innovation, which are essential for personal and societal development. However, recent studies have revealed that the mental health status of graduate students is far from optimistic (Xia et al., 2019). For example, the 2021 American College Health Association (ACHA) report highlighted depression as a significant issue affecting graduate students’ mental well-being (Wiesenthal et al., 2023). A survey of 2279 graduate students from 234 institutions across 26 countries revealed that 39% experienced moderate to severe depression, with graduate students being six times more likely to suffer from depression than the general population (Evans et al., 2018). It appears that graduate students worldwide are facing varying degrees of depression issues, though the causes of depression among graduate students in different countries may differ. Compared to those in the United States or European countries, Chinese graduate students are more likely to be affected by interpersonal factors that lead to depression. For instance, Confucian values that emphasize respect for teachers and encourage excessive effort, as well as the system of exam-oriented education, may all contribute to the depressive emotions experienced by Chinese postgraduates (Xiong et al., 2023). Therefore, it is particularly urgent to analyze the causes and mechanisms of depression among Chinese postgraduates. Such analysis is not only essential for providing a scientific foundation for future studies but is also crucial for enhancing the mental health of postgraduates.

### 1.1. Depression and Stress Levels

Depression is characterized as a shift in an individual’s emotional state from stable to depressed, accompanied by a range of symptoms, including loss of interest and cognitive sluggishness (Qian et al., 2019). In this study, “depression” refers specifically to depressive symptoms rather than clinically diagnosed major depressive disorder (MDD). Our focus is on self-reported depressive symptoms measured through standardized scales. Depression is a disorder in which negative affects and self-defeating behaviors are driven by erroneous beliefs and maladaptive information processing. Patients with depression often hold inaccurate and overly negative views of themselves, and it is characterized not only as an affective disorder but also as a ‘thought disorder’ that impacts cognition, behavior, and emotional well-being (Steven, 2022).

Stress is widely recognized as a key contributing factor to depression, as evidenced by numerous studies (Cheng & Li, 2022; Shen et al., 2018). Stress can reduce attention to positive stimuli (such as smiling faces) and disrupt emotional perception and regulation, thereby hindering positive self-perception (Peng et al., 2024). For example, some studies have found that exposure to different types of stress, especially chronic stress, is an important risk factor for adolescent depression (Stewart et al., 2024). Notably, stress from negative life events not only facilitates the rapid onset of depression but also exacerbates its long-term persistence (Lei et al., 2023).

In the educational context, research has found links between heavy academic stress and the development of depressive symptoms among students (Y. Liu et al., 2023). Focusing on the specific group of postgraduate students, the challenges they face are particularly complex, ranging from research tasks (e.g., paper publication and experimental studies) to peer competition, relationship management with supervisors, financial considerations, and work–life balance, each contributing significantly to their high-stress levels (Sun & Wang, 2021). The accumulation of such multiple sources of stress may not only lead to negative emotions but also seriously deplete graduate students’ motivation and work efficiency, subsequently triggering intensified emotional fluctuations, pessimism and disappointment, anxiety and depression, and even a significant decline in daily life management capabilities (Elamin et al., 2024). Therefore, this study proposes Hypothesis 1: Postgraduate students’ stress levels positively predict their depression.

### 1.2. The Mediating Role of Research Self-Efficacy

Cognitive mechanisms significantly influence the impact of stress on depressive mood (Bø et al., 2024), with self-efficacy being a pivotal factor. Empirical evidence has demonstrated an inverse relationship between stress and self-efficacy; as stress increases, self-efficacy tends to decrease (Hayes et al., 2020). This relationship is particularly obvious in educational contexts, where excessive academic stress can lead to reduced self-efficacy (Kristensen et al., 2023). Low self-efficacy and negative self-assessments can lead to negative emotional states; individuals who harbor self-doubt and perceive difficulty in achieving their goals are at a higher risk of developing depression (Pu et al., 2017). Research self-efficacy extends the concept of self-efficacy to the realms of academic and scientific research. It is essential for graduate students to conduct their research effectively, which is a statistically significant predictor of research success (Forester et al., 2004).

On one hand, high stress may lead to a decrease in research self-efficacy. Previous studies have shown that persistent psychological stress depletes graduate students limited mental resources, reducing their focus and engagement in research activities, ultimately undermining their research self-efficacy (Li et al., 2024). Moreover, studies have found that academic stress has a significant negative impact on research self-efficacy and can also reduce research self-efficacy by diminishing the research spirit (Hosseinabadi et al., 2023). On the other hand, research self-efficacy may also impact graduate students’ mental health. For example, a study among doctoral students at a medical university revealed a negative correlation between research self-efficacy and both depression and anxiety (C. Liu et al., 2019). Graduate students with low RSE and low confidence in their research are more likely to make negative attributions (e.g., some individuals engage in self-attribution, interpreting negative events as reflective of their personal flaws or inadequacies), which can exacerbate depressive symptoms (Bernstein et al., 2021). Consequently, this study posits Hypothesis 2: Research self-efficacy mediates the relationship between stress and depression among postgraduate students.

### 1.3. The Moderating Role of Growth Mindset

Mindset, as an implicit theory of how individuals perceive the plasticity of their traits (Fixed vs. Growth), profoundly influences cognitive and behavioral responses. Those with a more fixed mindset believe that individual traits such as intelligence, personality, and morality are stable, while those with a growth mindset believe that these traits are malleable and can be developed and enhanced through effort (Mouratidis et al., 2017). Research has explored the mechanisms through which a growth mindset benefits mental health. Beck’s Cognitive Theory of Depression indicates that thinking and beliefs are the main influences on emotional states (Pössel & Smith, 2020), thinking patterns affect the individual’s understanding and judgment of information in unfavorable environments, resulting in different emotional and behavioral responses, and many psychological problems can be ameliorated by changing an individual’s cognition. Yeager and Dweck (2023) propose that mindsets construct meaning systems for interpreting challenges and adversity. Individuals with a growth mindset view challenges as learning opportunities and perceive them as temporary obstacles, which reduces the risk of depression. Numerous global studies, particularly within East Asian cultures such as China, Japan, and Korea, have underscored the long-term positive effects of a growth mindset on mental health (Gouëdard, 2021).

Existing studies show that growth mindset interventions can reduce adolescents’ negative evaluations of stress (Yeager et al., 2022). People with a growth mindset are more likely to use effective coping strategies and are positively correlated with adaptive behaviors (Burnette et al., 2020). Moreover, scientific evidence has established that a growth mindset acts as a psychological buffer, mitigating the adverse effects of stress exposure and reducing susceptibility to depression and anxiety. Higher levels of a growth mindset encourage individuals to adapt to challenges, which emerges as a key mechanism through which a growth mindset promotes psychological well-being (Chang et al., 2024). Consequently, this study advances Hypothesis 3: The growth mindset exerts a moderating influence on the mediating pathway between postgraduate stress and depression.

**Figure 1:**
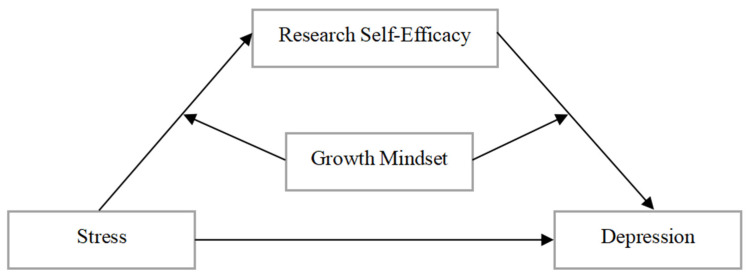
Hypothesized model of postgraduate stress affecting depression.

In summary, the present study proposes to construct a moderated mediation model (see Figure 1) to explore the effects of postgraduate student stress levels on depression and to examine the mediating role of research self-efficacy and the moderating role of the growth mindset.

## 2. Methods

### 2.1. Participants

The questionnaire was sent out electronically via the Wenjuanxing platform, which is the major online questionnaire platform in China and is widely used for academic study. The participants were graduate students (enrolled in master’s and doctoral programs) from 220 universities across 188 cities in 30 provinces of China. A total of 2767 anonymous responses were collected. Following a rigorous data cleaning process to eliminate invalid responses, a total of 2278 valid responses were retained, corresponding to an effective response rate of 82.33%. Demographically, the respondents comprised 1114 males (48.9%) and 1164 females (51.1%). In terms of academic level, there were 1335 master’s degree students (58.6%) and 943 doctoral degree students (41.4%). Regarding student status, 1464 were full-time students (64.3%) and 814 were part-time students (35.7%).

### 2.2. Instrument

Mindset Scale. The study employed Dweck’s Mindset Scale, which encompasses two dimensions: growth mindset and fixed mindset (Dweck, 2013). For the purposes of this research, the four positive questions of growth mindset in the scale were taken to measure. These items, totaling four items, were evaluated using a 5-point Likert scale, with responses ranging from “very much not in line with” to “very much in line with”, corresponding to scores of 1 to 5, respectively. The scale’s scoring was calibrated on a 5-point Likert scale, with descriptors from “very inconsistent” to “very consistent”, where higher scores suggest a stronger endorsement of a growth mindset by the student. The Cronbach’s *α* coefficient for this scale in this measurement was 0.77. Validated factor analysis data showed the following: *χ*^2^/df = 3.852, GFI = 0.998, IFI = 0.998, TLI = 0.993, CFI = 0.998, RMSEA = 0.035, SRMR = 0.010.

Depression–Anxiety–Stress Scale. The Depression–Anxiety–Stress Scales (DASS), developed by Clark and Watson, were utilized to measure self-reported depression and stress. The DASS comprises three sub-scales: the Depression Scale, the Anxiety Scale, and the Stress Scale, encompassing a total of 20 items (Clark & Watson, 1991). For the purposes of this investigation, only the Depression and Stress subscales were employed, each consisting of 7 items. The Stress Scale was further divided into 5 dimensions. Responses were recorded on a 5-point Likert scale, with scores ranging from 1 (“very non-compliant”) to 5 (“very compliant”), where higher scores indicate greater severity of depression or stress. The Cronbach’s *α* coefficients for the Depression and Stress scales were calculated to assess internal consistency, yielding values of 0.91 and 0.90, respectively, indicating high reliability for both scales. Validated factor analyses of the depression scale showed the following: *χ*^2^/df = 0.520, GFI = 0.999, IFI = 1.001, TLI = 1.001, CFI = 1.000, RMSEA = 0.000, SRMR = 0.004. Stress Scale = 0.004. Validated factor analysis data for the Stress Scale showed the following: *χ*^2^/df = 1.790, GFI = 0.997, IFI = 0.999, TLI = 0.998, CFI = 0.999, RMSEA = 0.019, SRMR = 0.009.

Research Self-Efficacy Scale. The Research Self-Efficacy Scale, developed by Black et al. (2013), was utilized in this study. This scale comprises two dimensions, encompassing a total of seven items: one dimension pertains to research methodology and writing communication, while the other focuses on research specification and organizational skills (Black et al., 2013). Responses were recorded using a 5-point Likert scale, with scores ranging from 1 (“very non-compliant”) to 5 (“very compliant”), where higher scores denote greater research self-efficacy. The Cronbach’s *α* coefficient of the scale was 0.91 in this measurement. Validated factor analysis data showed the following: *χ*^2^/df = 2.115, GFI = 0.996, IFI = 0.998, TLI = 0.997, CFI = 0.998, RMSEA = 0.022, SRMR = 0.009.

### 2.3. Analysis

Descriptive statistical analysis was conducted using SPSS 27.0, which was chosen for its robust capabilities in handling large datasets and a comprehensive suite of statistical procedures suitable for social science research. This analysis included tests for common method bias, independent samples *t*-tests, one-way ANOVA, and Pearson’s correlation analysis on the collected data (mean ± standard deviation). Additionally, the questionnaire underwent confirmatory factor analysis using AMOS 24.0, which was selected for its specialized features in structural equation modelling and its seamless integration with SPSS data files. AMOS’s graphical interface and advanced capabilities in handling complex moderated mediation models made it particularly suitable for testing our theoretical framework.

## 3. Results

### 3.1. Common Method Bias Test

Unrotated exploratory factor analyses were conducted using Harman’s single-factor test to assess the measured variables of stress, depression, growth mindset, and research self-efficacy. The results of the unrotated principal component factor analysis revealed four factors with eigenvalues exceeding 1. The first factor accounted for 36.18% of the variance, which is below the critical threshold of 40%. This indicates that the study is not significantly affected by common method bias (Zhou & Long, 2004).

### 3.2. Comparison of Variable Scores Across Demographic Characteristics

Independent samples *t*-test and one-way ANOVA were used to compare differences in scores on demographic variables for graduate student stress, research self-efficacy, depression, and growth mindset. As can be seen in Table 1, the differences in scores for stress were not statistically significant for sex, mode of study, or degree type, and there were statistically significant differences in grade level and scholarship attainment, with doctoral students (2.33 ± 0.9) having significantly higher stress scores than master’s students (2.09 ± 0.81), and post-hoc tests found that graduate students without scholarships had significantly higher stress scores than the rest of the graduate students.

Research self-efficacy did not have statistically significant differences in scores for sex, mode of study, or degree type, and there were statistically significant differences in grade level and scholarship acquisition; the research self-efficacy scores of master’s students (4 ± 0.84) were significantly higher than those of doctoral students (3.71 ± 0.93), and the post-hoc test found that the research self-efficacy scores of provincial and municipal scholarship recipients were significantly higher than those of other postgraduate students.

There was no statistically significant difference in the scores of depression in terms of sex, modes of study, or types of degree; there was a statistically significant difference in terms of grade level and scholarship acquisition, the depression scores of doctoral students (2.41 ± 0.92) were significantly higher than those of master’s students (2.3 ± 0.88), and post-hoc tests found that the depression scores of graduate students without scholarships were significantly higher than those of other graduate students.

There was no statistically significant difference in growth mindset scores by sex, mode of study, or type of degree, and there was a statistically significant difference in grades and scholarships received, with master’s students (3.75 ± 0.84) scoring significantly higher than doctoral students (3.43 ± 0.84), and post-hoc tests found that provincial and municipal scholarship recipients scored significantly higher than other graduate students.

### 3.3. Descriptive Statistics and Correlation Analysis of Variables

Descriptive statistics and correlation analyses were conducted for each variable, with the results presented in Table 2. The analysis revealed a statistically significant positive correlation between stress and depression (*r* = 0.403, *p* < 0.01). Conversely, stress exhibited statistically significant negative correlations with both research self-efficacy (*r* = −0.430, *p* < 0.01) and growth mindset (*r* = −0.340, *p* < 0.01). Additionally, research self-efficacy was found to have a statistically significant positive correlation with growth mindset (*r* = 0.394, *p* < 0.01), while it showed a statistically significant negative correlation with depression (*r* = −0.426, *p* < 0.01). Similarly, a growth mindset was significantly negatively correlated with depression (*r* = −0.347, *p* < 0.01).

### 3.4. Moderated Mediation Effect Test

First, structural equation modeling was constructed using AMOS 24.0. Bootstrap tests were performed, and 5000 repeated samples and 95% confidence intervals were set. Items were item-packed for postgraduate stress and depression using factor loading balance to test the direct effect of postgraduate stress on depression. The model fit was good: *χ*^2^/df = 2.022, GFI = 0.996, IFI = 0.998, TLI = 0.998, CFI = 0.998, RMSEA = 0.021, and SRMR = 0.017. The path coefficient between stress to depression was *β* = 0.22, 95%, CI [0.41, 0.52], *p* < 0.001, indicating that postgraduate students’ stress significantly and positively predicted their depression.

Second, the mediating variable research self-efficacy was added to establish a mediation model, and research self-efficacy was item-packed using the factor loading balance method to test its mediating effect. The model fitted well: *χ*^2^/df = 2.140, GFI = 0.992, IFI = 0.997, TLI = 0.996, CFI = 0.997, RMSEA = 0.022, SRMR = 0.016. The coefficient of the path of stress to research self-efficacy was *β* = −0.24, *p* < 0.001, and the path of research self-efficacy to depression was *β* = −0.222, *p* < 0.001. Bootstrap test results showed that the mediating effect value of research self-efficacy was 0.17, 95% CI [0.13, 0.20], and the total effect value was 0.46, which accounted for 35.6% of the total effect, suggesting that research self-efficacy partially mediates the relationship between postgraduate stress and depression.

**Figure 2:**
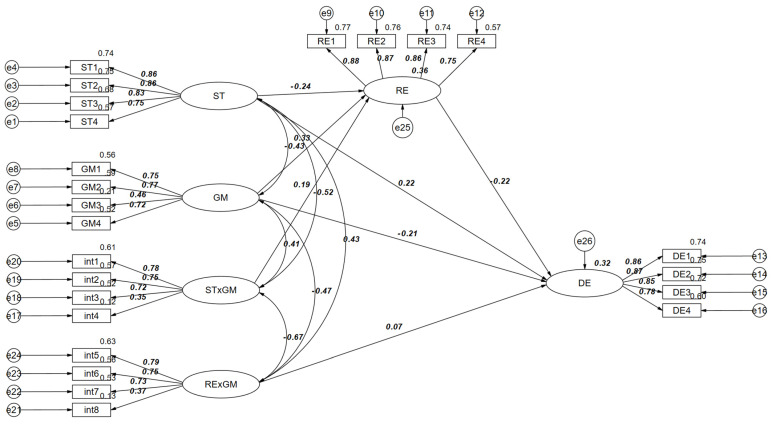
Path diagram of the moderated mediation model.

Again, the mediated model was constructed with moderation by adding the moderating variable growth mindset to the mediated model (see Figure 2). The model fitted well: *χ*^2^/df = 6.663, GFI = 0.947, IFI = 0.954, TLI = 0.946, CFI = 0.953, RMSEA = 0.050, SRMR = 0.050. Bootstrap test results showed that the interaction term of stress and growth mindset was a statistically significant predictor of research self-efficacy (*β* = 0.189, *p* < 0.001, 95% [0.11, 0.26]), suggesting that growth mindset plays a moderating role between stress and research self-efficacy and that the interaction term between research self-efficacy and growth mindset was a statistically significant predictor of depression (*β* = 0.068, *p* < 0.05, 95% [0.00, 0.13]), suggesting that the growth mindset plays a role in the moderating role between research self-efficacy and depression (see Table 3 for specific pathways).

Analyzing the mediating role of scientific research self-efficacy between stress and depression at different levels of growth mindset, it was found that the mediating pathway role of stress affecting depression through scientific research self-efficacy was not statistically significant when the level of growth mindset was high (*β* = −0.005, *p* > 0.05, 95% [−0.05, 0.01]) and that the mediating pathway role of stress affecting depression through scientific research self-efficacy was statistically significant when the level of growth mindset was average (*β* = 0.053, *p* < 0.001, 95% [0.03, 0.08]). The stress-mediated pathway effect of depression through research self-efficacy was statistically significant (*β* = 0.053, *p* < 0.001, 95% [0.03, 0.08]). When growth mindset was at a low level, the stress-mediated pathway effect of depression through research self-efficacy was statistically significant (*β* = 0.167, *p* < 0.001, 95% [0.11, 0.26]). Further comparative analysis showed the following: The difference in moderated mediation effects between high and low growth mindset groups was *β* = −0.171, *p* < 0.001, 95% CI [−0.28, −0.10]; the difference between high and middle growth mindset groups was *β* = −0.058, *p* < 0.01, 95% CI [−0.10, −0.04]; and the difference between middle and low growth mindset groups was *β* = −0.113, *p* < 0.001, 95% CI [−0.21, −0.06] (see Table 4). The results indicate that when growth mindset levels are lower, the impact of graduate students’ stress on depression through research self-efficacy is stronger.

## 4. Discussion

### 4.1. The Variety of Postgraduate Student Stress and Their Impacts on Depression

This study identified graduate student stress as a statistically significant positive predictor of depression, indicating that higher levels of stress are associated with more severe depressive symptoms. This finding corroborates existing research on the stress–depression relationship, reinforcing stress as a pivotal risk factor of depression (Lemoult et al., 2023). Stress, as an environmental variable, contributes to depression through various interacting factors. These include the negative emotional valence inherent in stressful events, which directly elicits negative emotional responses such as anxiety, and the impact of stress on individual cognition, leading to negative self-perceptions and views of the external world, thereby indirectly compromising emotional stability. For graduate students, common stressors include time pressure, failures in publishing papers, inadequate preparation for academic conferences, and fear of poor academic performance, which are almost universally encountered during their academic career. This is especially true for doctoral students, who must independently determine their research direction, produce innovative results, and ensure the quality of their dissertations (J. Jiang, 2023). Such accumulated stress can not only diminish graduate students’ research self-efficacy but also precipitate depressive symptoms.

Moreover, this diverse range of influencing factors is cross-cultural. Graduate students in both China and Western countries face similar pressures, such as the stress of academic failure, issues in the relationship between graduate students and supervisors, and concerns over the innovation of academic achievements. However, the weight of individual factors may vary in different cultural contexts. Therefore, we believe that the findings of this study can also inspire other countries to appropriately address the mental health issues of graduate students. Therefore, a comprehensive understanding of the stress faced by graduate students and effective strategies for managing it are crucial for universities around the world.

### 4.2. Research Self-Efficacy Determines How Graduate Students Cope with Stress

The current study ascertained that research self-efficacy serves as a partial mediator between stress and depression among graduate students. This implies that the stress levels experienced by graduate students are not only directly predicted by depression but also exert an indirect influence on depressive symptoms via the mediating role of research self-efficacy.

Initially, the empirical findings of this study reveal a statistically significant negative correlation between the stress levels of graduate students and their research self-efficacy. This correlation corroborates extant literature suggesting that personal academic accomplishments, such as the attainment of senior academic titles and a substantial record of scholarly publications, are closely linked to research self-efficacy (Griffioen et al., 2013). In contradistinction, graduate students who encounter obstacles or achieve limited outcomes in their research are more likely to experience stress. Excessive psychological stress depletes mental resources and disrupts time management and task focus (Li et al., 2024). Such experiences can undermine their confidence in their research capabilities, precipitating a decline in research self-efficacy and thereby cause a negative cognitive feedback loop. Furthermore, the quality of the supervisory relationship, identified as a salient stressor for graduate students, exerts both direct and indirect effects. It directly influences the trajectory of research self-efficacy and indirectly impacts the psychological well-being of graduate students, manifesting in increased anxiety levels (Xie et al., 2023).

Subsequently, the study revealed a statistically significant and negative predictive relationship between graduate students’ research self-efficacy and depression. Drawing upon cognitive motivation theory, it is posited that graduate students with a low sense of research self-efficacy are predisposed to doubt their competencies when confronted with research-related challenges. They are more inclined to adopt avoidance strategies or give up. Even in instances where they attempt to solve problems, a deficiency in confidence leads to an overestimation of problems and an underestimation of their problem-solving capabilities (Wang & Liu, 2002). Moreover, Graduate students with low research self-efficacy tend to overly focus on the negative aspects of research stress, concerning its potential detrimental impact on their health. This excessive preoccupation with negative outcomes further precipitates the onset of negative emotions, including anxiety and depression. As stress accumulates and research self-efficacy progressively deteriorates, the likelihood of developing depressive symptoms escalates, thereby posing a statistically significant threat to the holistic physical and mental health of graduate students.

### 4.3. A Growth Mindset Serves as a Barrier in the Pathway of Stress Effects on Depression

The present study examined the individual variances in graduate students’ depressive response to stress, with particular emphasis on the growth mindset as a salutary mechanism capable of mitigating the pathway through which stress impacts depression via research self-efficacy. This finding supports the diathesis-stress model of depression, which indicates that certain individuals harbor psychological susceptibilities that are readily triggered by stress, whereas those devoid of such vulnerabilities remain psychologically unscathed when encountering similar stressful events (Chen et al., 2011). Specifically, when graduate students exhibit lower levels of growth mindset, the inverse relationship between stress and research self-efficacy becomes more pronounced, and their depression scores are significantly increased in comparison to students with higher levels of growth mindset. This observation is consonant with the hopelessness theory of depression, which posits that individuals are more likely to experiencing negative emotions when they attribute their personal inadequacies as the reason for negative life events (Zhang et al., 2024).

Conversely, graduate students with higher levels of growth mindset demonstrate a heightened degree of resilience. In the face of stress, they experience a diminished negative influence from pressure on research self-efficacy, and this protective effect further attenuates the association between research self-efficacy and depression. The salutary role of the growth mindset is predicated on its foundational belief that one’s abilities are malleable and can be enhanced through sustained effort. This epistemological stance not only preserves their research self-efficacy but also effectively curtails the escalation of depressive symptoms (Y. Jiang et al., 2019). However, according to previous studies, the growth mindset itself is also influenced by a variety of factors. Therefore, there may be many other factors that simultaneously moderate the relationship between stress and depression. For example, the Chinese context of this study may introduce Confucian values that emphasize hard work, which is an important influencing factor of the growth mindset and may also independently moderate the relationship between stress and depression. Therefore, there are more potential confounding variables that are worth further investigation.

### 4.4. Practical Implications

According to the current findings, research self-efficacy and growth mindset of graduate students both play mediating roles in the relationship between stress and depression. Therefore, universities and supervisors should address the mental health issues of graduate students from multiple aspects simultaneously. First, compulsive mental health training should be conducted for supervisors, and their responsibilities in student mental health should be clarified. Furthermore, an equal and trusting relationship should be encouraged between supervisors and students. Second, the universities should optimize the academic environment. The research tasks and academic workload should be maintained at an appropriate level, and the institute should provide academic resources and research guidance to enhance their academic self-efficacy. Finally, it is important to cultivate a growth mindset in graduate students. Support groups and academic salons could be built to encourage graduate students to share and discuss learning difficulties and develop their resilience.

### 4.5. Limitations and Suggestions for Future Studies

Firstly, the study utilized a cross-sectional survey design, which limits the ability to capture the dynamic changes of how stress, research self-efficacy, and growth mindset among graduate students affect depressive symptoms. Thus, we are looking forwards to future studies that employ a longitudinal research method to explore the changes in stress, depression, research efficacy, and growth mindset among graduate students.

Secondly, the investigation focused solely on the relationship between graduate student stress and depression through the lens of individual characteristics, neglecting the potential influence of environmental factors. In particular, how aspects of the Asian culture, such as respecting teachers and working hard, affects postgraduate students’ depression and growth mindset is missing. Future research should consider incorporating a broader range of variables, including environmental and cultural factors, to provide a more comprehensive understanding of the phenomenon.

Third, the questionnaire was implemented in a self-reporting format, which may introduce potential issues such as social desirability bias, interpretation bias, and subjectivity. Therefore, we anticipate that future research will employ diverse research designs, such as longitudinal designs, quasi-experimental studies, and qualitative research, to verify the results of this study.

## 5. Conclusions

In the context of prevalent mental health issues among Chinese graduate students, this study investigates the correlation between stress and depression, with a focus on the mediating role of research self-efficacy and the moderating influence of a growth mindset. The findings reveal that research self-efficacy significantly mediates the relationship between stress and depression. Additionally, a growth mindset was found to moderate both pathways in the stress–research self-efficacy–depression nexus.

## Figures and Tables

**Table 1:** Comparison of scores on demographic variables for stress, research self-efficacy, depression, and growth mindset (*M* ± *SD*) (*N* = 2278).

	Variant	Stress	Research Self-Efficacy	Depression	Growth Mindset
Sex	men	2.17 ± 0.83	3.9 ± 0.86	2.33 ± 0.9	3.61 ± 0.87
	women	2.21 ± 0.88	3.87 ± 0.91	2.35 ± 0.9	3.62 ± 0.85
	*t*	−1.00	0.94	−0.53	−0.22
Grade	Master	2.09 ± 0.81	4 ± 0.84	2.3 ± 0.88	3.75 ± 0.84
	Doctor	2.33 ± 0.9	3.71 ± 0.93	2.41 ± 0.92	3.43 ± 0.84
	*t*	−6.64 ***	7.74 ***	8.81 ***	−2.92 **
Mode of study	full-time	2.2 ± 0.85	3.9 ± 0.86	2.34 ± 0.88	3.62 ± 0.83
	part-time	2.17 ± 0.87	3.86 ± 0.94	2.35 ± 0.94	3.62 ± 0.91
	*t*	0.696	0.918	−0.197	0.078
Scholarships awarded	National Scholarship	2.29 ± 0.88	3.71 ± 0.94	2.41 ± 0.92	3.46 ± 0.92
	Provincial/Municipal Scholarship	2.07 ± 0.8	4.01 ± 0.83	2.28 ± 0.84	3.71 ± 0.83
	University Scholarship	2.27 ± 0.88	3.81 ± 0.93	2.39 ± 0.95	3.6 ± 0.86
	No scholarships	2.59 ± 0.97	3.67 ± 0.84	2.49 ± 1.04	3.42 ± 0.81
	*F*	21.2 ***	16.04 ***	4.24 **	10.85 ***

Note: ** *p* < 0.01, *** *p* < 0.001.

**Table 2:** Descriptive statistics and correlation analysis of variables (*N* = 2278).

	*M*	*SD*	1	2	3	4
1. Stress	2.190	0.857	1			
2. Research self-efficacy	3.883	0.889	−0.430 **	1		
3. Depression	2.343	0.899	0.403 **	−0.426 **	1	
4. Growth mindset	3.619	0.856	−0.340 **	0.394 **	−0.347 **	1

Note: ** *p* < 0.01.

**Table 3:** Path coefficients between variables.

Path	*β*	S.E.	C.R.	Boot CI Lower Limit	Boot CI Upper Limit
Stress → Depression	0.22	0.027	8.793 ***	0.163	0.283
Stress → research self-efficacy	−0.24	0.028	−9.341 ***	−0.240	−0.301
Research self-efficacy → depression	−0.222	0.027	−8.204 ***	−0.288	−0.155
Growth mindset → research self-efficacy	0.326	0.028	12.493 ***	0.258	0.391
Growth mindset → depression	−0.206	0.03	−7.367 ***	−0.268	−0.140
Stress × growth mindset → research self-efficacy	0.189	0.059	6.433 ***	0.113	0.258
Research self-efficacy × growth mindset → depression	0.068	0.05	2.476 *	0.004	0.134

Note: * *p* < 0.05, *** *p* < 0.001.

**Table 4:** Mediating role of research self-efficacy at different levels of growth mindset.

	Standardized Effect Value	Boot CI Lower Limit	Boot CI Upper Limit	*p*
High Score Group	−0.005	−0.045	0.014	0.446
Middle Score Group	0.053	0.033	0.078	0.000
Low Score Group	0.167	0.108	0.261	0.000
High Score Group–Low Score Group	−0.171	−0.275	−0.1	0.000
High Score Group–Medium Score Group	−0.058	−0.099	−0.037	0.001
Medium Score Group–Low Score Group	−0.113	−0.212	−0.056	0.000

## Data Availability

The data presented in this study are available from the corresponding author upon request.
